# "I expected little, although I learned a lot": perceived benefits of participating in HIV risk reduction sessions among women engaged in sex work in Uganda

**DOI:** 10.1186/s12905-022-01759-1

**Published:** 2022-05-13

**Authors:** Ozge Sensoy Bahar, Proscovia Nabunya, Josephine Nabayinda, Susan S. Witte, Joshua Kiyingi, Larissa Jennings Mayo-Wilson, Prema Filippone, Lyla Sunyoung Yang, Janet Nakigudde, Yesim Tozan, Fred M. Ssewamala

**Affiliations:** 1 Brown School, Washington University in St. Louis, One Brookings Drive, St. Louis, MO 63130 USA; 2 International Center for Child Health and Development, Brown School, Washington University in St. Louis, St. Louis, MO USA; 3 International Center for Child Health and Development Field Office, Masaka, Uganda; 4 Columbia University School of Social Work, New York City, NY USA; 5 School of Global Public Health, University of Carolina Chapel Hill, Chapel Hill, NC USA; 6 College of Health Sciences, Makerere University, Kampala, Uganda; 7 School of Global Public Health, New York University, New York City, NY USA

**Keywords:** Women engaged in sex work, HIV risk reduction, Commercial sex work, Qualitative, Behavioral interventions, Sub-Saharan Africa

## Abstract

**Background:**

The global HIV burden remains a public health concern. Women engaged in sex work (WESW) are at higher risk of acquiring HIV compared to the general adult population. Uganda reports high rates of HIV prevalence among WESW. While WESW in Uganda have long been the subject of surveillance studies, they have not been targeted by theory-informed HIV prevention intervention approaches. In this study, we explored the perceived benefits of an evidence-based HIV risk reduction intervention that was implemented as part of a combination intervention tested in a clinical trial in Uganda.

**Methods:**

As part of a larger randomized clinical trial, we conducted semi-structured in-depth interviews with 20 WESW selected using a stratified purposive sampling. All interviews were conducted in Luganda, language spoken in the study area, and audio-recorded. They were transcribed verbatim and translated to English. Thematic analysis was used to analyze the data.

**Results:**

WESW’s narratives focused on: (1) condom use; (2) alcohol/drug consumption; (3) PrEP use; (4) “handling” customers; and (5) "massaging” customers. WESW agreed that male condom was one of the important learning points for them and planned to continue using them while female condoms were received with mixed reactions. Many women appreciated receiving information about the risks of consuming alcohol and drugs, and discussed how they reduced/ eliminated their consumption. PrEP information was appreciated though identified by fewer WESW. Handling a client was discussed as a helpful strategy for safer sex through improved ability to convince customers to use condoms or avoiding sex. Massaging was also beneficial to avoid penetrative sex, but similar to female condom, massaging also yielded mixed perceptions.

**Conclusion:**

WESW found the intervention beneficial and described ways in which it improved their ability to engage in safer sex and stay healthy. The fact that WESW identified other strategies beyond condom use as helpful underlines the importance of adopting a comprehensive approach to behavioral interventions targeting HIV prevention even when combined with other interventions. Additionally, WESW’s narratives suggest that incorporating the tenets of social cognitive theory and harm reduction approaches in HIV prevention among this population can result in risk behavior change.

**Supplementary Information:**

The online version contains supplementary material available at 10.1186/s12905-022-01759-1.

## Background

The global HIV burden remains a critical public health concern, with an estimated 37.9 million people living with the disease and 1.7 million newly diagnosed in 2018 [1]. More than half (62%) of all new HIV infections are among key populations and their sexual partners, including women engaged in sex work (WESW) [2]. Key populations are at significantly higher risk of acquiring HIV, estimated at 13–30 times higher compared to the general adult population [2]. Similar to other countries with high HIV burden in sub-Saharan Africa (SSA), Uganda reports high rates of HIV prevalence among WESW, estimated at 37% and accounting for 18% of all new infections in the country [3].

Various social, cultural, political, and economic factors influence WESW’s heightened risk for contracting HIV. WESW may have limited bargaining power to negotiate safe sex practices due to higher premiums offered for unprotected sexual acts, increasing their vulnerability to HIV infection [4–7]. Studies from SSA show that WESW experience intense stigma, discrimination, and consequent social marginalization, which in turn deepens their vulnerability to HIV infection [8–10] and interferes with their ability to engage with HIV and sexually transmitted infections (STIs) prevention and treatment services [8, 11]. Thus, effective HIV prevention interventions require combining approaches that target all risk factors that increase transmission risks as well to improve HIV care and treatment outcomes among WESW.

Several studies have evaluated the efficacy of HIV risk reduction intervention efforts among WESW in SSA. For example, studies have documented consistent increases in self-reported condom use among WESW with paying partners due to improved self-efficacy in condom negotiations skills [12, 13] Effective strategies included condom promotion using peer educators, condom distribution and risk reduction, distribution of education materials, and counseling sessions on HIV testing and risk reduction with program staff [13, 14].

However, promoting condom use-only strategies, while important, may not be sufficient to reduce HIV transmission among WESW. A review of effective combination interventions targeting HIV risk behaviors and HIV continuum of care outcomes for WESW in SSA indicate that interventions that combined structural, biomedical and behavioral strategies tend to accumulate the desired outcomes, including behavioral change communication, utilization of health facilities, reduction in sexual violence, alcohol and substance use—all of which increase the risk of HIV transmission among WESW [15]. Another study evaluated HIV prevention intervention efforts that included community-based outreach, HIV and substance use education and information, as well as safer sex practices for substance using WESW in South Africa [16]. Study results documented a significant decrease in all substances used, number of sexual partners, and number of times engaged in sex work. Other effective interventions are those that were provided within geographical areas where WESW work and live [17]. Taken together, these findings indicate that multicomponent interventions, that utilize community-based efforts, may be more effective in HIV prevention and reducing risks among WESW, especially in low-resource settings.

In Uganda, as in all of SSA, WESW have long been the subject of surveillance studies, and HIV prevention programs have been limited to “information, education, and communication” or Behavior Change Communication type strategies that do not include behavioral theory-informed components [18]. WESW have not been consistently targeted by theory-informed, behavioral science-based, HIV prevention intervention approaches [19–22]. In addition, few studies have qualitatively examined WESW’s experiences with HIV risk reduction interventions and how they may benefit from them. Yet, qualitative data are critical for better tailoring HIV risk reduction and prevention interventions for WESW. Hence, in this article, we qualitatively examined the perceived benefits of an evidence-based, behavioral HIV risk reduction intervention delivered as part of a combination intervention among WESW in southwestern Uganda. To our knowledge, this intervention is among the first to be based on social cognitive theory, to incorporate intentional skills modeling, practice and generalizing through short- and longer-term goal setting and building social norms and support among WESW.

## Methods

This study, funded by the National Institute of Mental Health (R01MH116768) is a randomized clinical trial (NCT03583541) that evaluates the efficacy of adding economic empowerment components to traditional HIV risk reduction intervention to reduce new incidences of STIs and of HIV among WESW in HIV hotspots in the greater Masaka region of Uganda (see study protocol for more details [23]). Study sites were randomly assigned to three treatment conditions: (1) Control arm that received HIV risk reduction sessions focused on equipping participating women with the skills to reduce the spread of HIV; (2) Treatment arm 1 that received HIV risk reduction sessions combined with a matched savings account and financial literacy training with integrated behavioral economics principles, aimed at training participants on issues related to the importance of savings, banking services, budgeting, and debt management; and (3) Treatment arm 2 that received HIV risk reduction sessions, a matched savings account, financial literacy training, and vocational skills training and mentorship sessions to economically empower WESW to start up an income-generating activity [23]. Treatment arms 1 and 2 were combined under Treatment arm 1 due to COVID-19 that interfered with recruitment and data collection.

WESW were eligible for the study if they: (1) were 18+ years; (2) reported engagement in unsafe transactional sex (defined as a sex act in exchange for pay) in the past 30 days; and (3) reported engagement in one or more episodes of unprotected sex in the past 30 days [23].

The study used an embedded experimental mixed methods design [24] where qualitative data was collected across the two study arms at intervention completion (Time 1), at 12-month (Time 2) and 24-month (Time 3, to be collected) follow-up.

### Ethical considerations

All study procedures were approved by the Washington University in St. Louis Institutional Review Board (#201811106), Columbia University Institutional Review Board (IRB-AAAR9804) and the in-country Institutional Review Boards in Uganda: Uganda Virus Research Institute (UVRI—GC/127/18/10/690), and the Uganda National Council of Science and Technology (UNCST—SS4828). Written consent was obtained from all WESW in the study. The study is also overseen by an in-country Data Safety and Monitoring Board [23]. Participants who tested positive for HIV during the study were referred to HIV clinics for ART treatment and counseling.

### Study setting

HIV prevalence among adults in Uganda aged 15–49 years was estimated at 5.4% in 2020, similar to other sub-Saharan African countries with high HIV burden [25]. Uganda reports high rates of HIV prevalence among WESW, estimated at 37% and accounting for 18% of all new infections in the country [25]. The study is conducted in the Greater Masaka region, consisting of 7 political districts, across which the HIV prevalence among the general female population was %9.5 [25]. HIV prevalence among WESW in Rakai and Masaka districts was reported as high as 61% [26].

### HIV risk reduction intervention

Guided by social cognitive theory [27, 28] and a harm reduction approach [29, 30], the HIV risk reduction component of the Kyaterekera study focused on both sexual and drug/alcohol use risk reduction, and is designed to increase communication, problem-solving skills, and self-efficacy related to safe-sex behaviors and substance use. The intervention incorporated many of social cognitive theory’s social-cognitive mediators within the HIV prevention context, including male and female condom use, communication skills and building social support. One of the central tenets of social cognitive theory is self-efficacy. Self-efficacy is achieved by incorporating into sessions content on triggers for risk taking, communication skills to negotiate safer sex and substance behaviors, and goal setting to generalize new skills into everyday practice. Self-efficacy has been found to affect whether people intend to change their behavior, the degree of effort they invest in changing, and whether behavior change is maintained long-term [28]. Self-efficacy with respect to negotiating and using condoms with sexual partners –intimate or paying– has been found to be a strong predictor of condom use [31] and is often found in conjunction with empowerment in sexual relationship decision making [32]. The harm reduction approach is a well-accepted pragmatic approach to high-risk behaviors if abstinence from the behavior is not achievable [29, 30] and is infused throughout the curriculum.

**Fig. 1:**
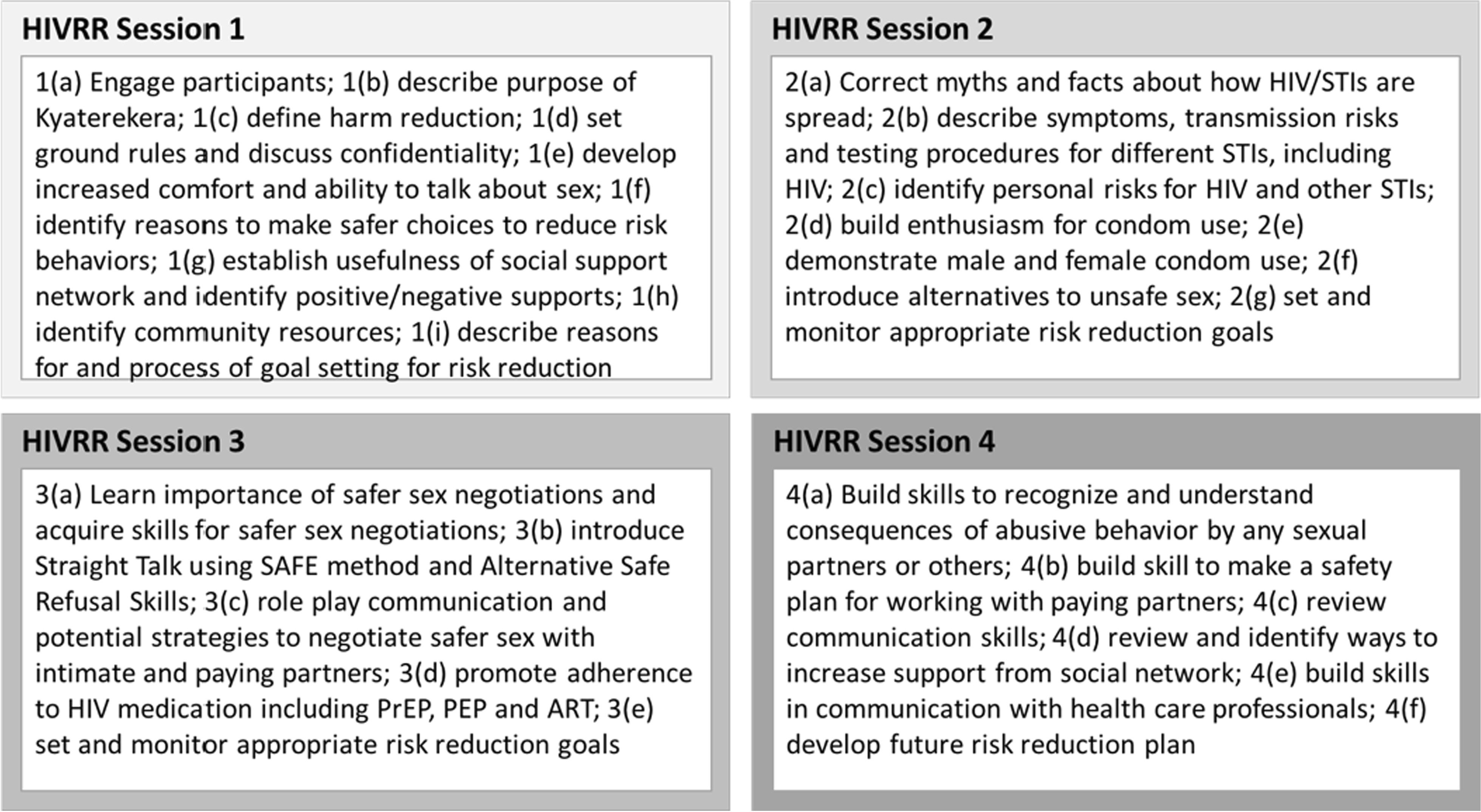
HIVRR session content

All women in the study received 4 sessions of an evidence-based, HIV/STI risk reduction intervention tested in three previous studies conducted in Central Asia [33, 34]. The manualized HIV risk reduction sessions were adapted to the Ugandan context (see Fig. 1), in consultation with two members of the Community Collaborative Board [35], who were WESW representatives. Specifically, they helped to infuse appropriate language, examples, activities, and lived experiences relevant to WESW in Uganda. The curriculum was translated and delivered in Luganda (local language spoken in the study area).

The sessions were facilitated by eight community health workers who have worked with the International Center on Child Health and Development on other studies in the same region. The community health workers participated in a one-week training conducted by one of the study principal investigators and a study consultant who has extensive experience in HIV risk reduction interventions. The 2-h sessions were delivered twice a week for 2 weeks in groups of 20–25 WESW. The session proceedings were supervised by research assistants. Additionally, fidelity assessments were completed at the end of each session for quality control checks.

Role plays were conducted to provide opportunities for women to practice skills covered during the sessions and to identify and problem-solve barriers to applying them. In addition, regular check-ins at the beginning of each session were conducted to ensure that WESW received the support and referrals they needed if they felt that their safety was threatened by an intimate partner or by other persons. In addition, participants were given information on available organizations officering linkage to HIV care, drug treatment, and other services in the study region. During the sessions, interested WESW were initiated on pre-exposure prophylaxis (PrEP) and male condoms were distributed throughout the intervention period.

### Qualitative sample selection and characteristics

The qualitative sampling was based on participants’ attendance of intervention sessions, i.e., HIV risk reduction (4 sessions) and financial literacy training (6 sessions). For each site, one participant from the highest, mid (average) and lowest quartiles of total number of attended sessions was selected (n = 57). For Time 1 qualitative data collection, semi-structured interviews were completed with 20 participants from only seven sites due to intervention delivery delays and lock-downs related to COVID-19. Participants’ characteristics are provided in Table 1.

**Table 1:** Participant demographics

Variable	Total sample [N = 20] %(n)
Age (min/max: 20–46)	31.45 (SD = 5.82)
Marital status	
Married/in relationship	30(6)
Single	5(1)
Others	65(13)
Level of education	
Primary school education	90(18)
Secondary school education	10(2)
Household composition (min/max: 1–6)	2.6 (SD = 1.31)
Age when first engaged in sex work (min/max:14–35)	23.95 (SD = 6.38)
Number of sex partners (min/max: 2–120)	31.75 (32.86)
HIV status	
Positive	50(10)
Negative	50(10)
STIs status	
Positive	15(3)
Negative	85(17)
HIVRR session attendance	
Session 1	90(18)
Session 2	65(13)
Session 3	60(12)
Session 4	75(15)

Participants’ age ranged between 20 to 46 years (mean age 31.45). Fifteen participants were from the control arm of the study (receiving only HIV risk reduction sessions) and 5 from the treatment arm. Seven participants were sampled in the low attendance, six in average attendance, and seven in high attendance groups from across seven sites. Thirty percent of the participants were married or in a relationship, 90% had primary school education and 10% secondary education. Fifty percent tested HIV positive and 15% tested positive for any of the STIs (Chlamydia, Gonorrhea and Trichomonas). The average age at sex work debut was about 24 and the average number of sex partners within the last 30 days was 32. To maintain the privacy and confidentiality of study participants, pseudonyms are used throughout the article.

### Qualitative data collection

Face-to-face semi-structured in-depth interviews were conducted following intervention completion (Time 1) and 12-month (Time 2) follow-up and will be conducted again at 24-month (Time 3) follow-up. In this article, we report findings from Time 1 qualitative interviews (n = 20). Data from 17 participants was collected in December 2019. The remaining 3 interviews were completed in December 2020 as a result of delays in intervention completion at those specific sites due to COVID-19. The Time 1 interviews focused on: (1) participants’ experiences with the respective intervention and its specific components (i.e., HIV risk reduction, savings and financial literacy); (2) key multi-level (individual, economic, family, contextual, and programmatic) influences that affected their participation; and (3) perceptions on intervention sustainability. Interview questions used for the purpose of this article included: “What content covered in the sessions has been most helpful to you?”; “How have you been using them in your everyday life?”; “What content covered in the sessions has been least helpful to you? Why?”; and “What content do you foresee yourself continuing to use in the future? How?” (see Additional file 1).

Interviews were conducted in Luganda, the widely spoken language in the study region. Interview questions were translated (English to Luganda) and back-translated by two proficient team members. Questions were reviewed and revised by the study team that included research assistants and co-investigators fluent in both languages to ensure that they sounded natural and conversational, and conveyed the same meanings intended. Interviews were conducted by male and female research assistants fluent in both Luganda and English and trained extensively by two authors with qualitative research expertise. Interviews lasted between 38 to 100 min (mean = 60 min) and were conducted in a private place with only the research assistant and the participant present. All interviews were audio taped and field notes were taken upon completion of the interview.

### Qualitative data analysis

Interviews were first transcribed verbatim and then translated from Luganda to English by research assistants fluent in both languages. Dedoose analytic software was used for data analysis. We used inductive techniques for thematic analysis of the data [36–38]. Thematic analysis allowed us to use preexisting themes, such as male condom use, alcohol consumption but also derived subthemes that emerged from the data, such as “feeling empowered” and quitting alcohol. Interview transcripts were initially read multiple times and independently coded by three authors using sensitizing concepts informed by the existing literature and the content of the intervention as well as identifying emergent themes (open coding) [39]. Broader themes were broken down into smaller, more specific units until no further subcategory was necessary. Initial codes were discussed during team meetings and reorganized when necessary to create a final codebook that was used to code all transcripts.

The secondary analysis, conducted by two authors, compared and contrasted themes and categories to identify similarities, differences, and relationships among findings. Peer debriefing, member checking, and audit trail were used to ensure rigor [40, 41]. The codes and the findings were presented to two members of the research team who were not involved in the data analysis to discuss the plausibility of themes and related findings [40]. Moreover, member checking was used where the study findings were shared with the study’s Community Collaborative Board that includes representatives of the WESW across seven districts. As a result of the consultation with the Community Collaborative Board, the terminology originally translated as “romancing” was changed to “massaging” as WESW stated that that is the wording they used to refer to non-penetrative sex.

## Results

As described above, the HIV risk reduction sessions covered a range of information, including HIV and STIs as well strategies to prevent one from acquiring or spreading diseases that are sexually transmitted. Participating WESW were asked about what they found most beneficial about the content covered in the sessions and how they applied it in their everyday lives. In addition, participants were also asked about content they found least helpful. WESW’s narratives focused on five themes: 1. Condom use; 2. Alcohol/drug consumption; 3. PrEP use; 4. “Handling” customers; and 5. "Massaging” customers.

### Condom use

#### Male condom use

Majority of WESW talked about how useful the content they received on male condoms was beneficial to them. Learning that condoms would prevent them from contracting STIs, and HIV/AIDS was the main motivator for them as they planned to continue using condoms. WESW appreciated both the information about the benefits of using male condoms as well as the skills taught around how to put a male condom on and convince the customers to wear one. For instance, Grace, engaged in sex work for about five years, mentioned that in addition to learning how to put condoms on, it was critical for her to learn that condoms may protect someone from spreading or contracting HIV. This is particularly important as she was one of the participants who reported a much higher number of sex partners within the last 30 days. She also underlined how helpful it was for the program to distribute condoms as she discussed the challenges related to securing condoms.

Grace: We received condoms from Kyaterekera…. They helped us and supplied us with condoms because sometimes we do not have them, sometimes I go to the health facility or clinic where we buy them, sometimes you find the condoms that are available have expired! It is such a relief that you provide condoms to us and showing us how to put on a condom, most of us do not know how to put on a condom… And now that I can appropriately use a condom, and I know that I have HIV, I do not want to infect another person! I learned that it is not good to infect other people.

Relatedly, Ruth, engaged in sex work for four years, stated that the HIV risk reduction sessions taught them how to stay safe when engaging in sex work, including how men can safely wear a condom.

Ruth: We risk our lives in engaging in sex work thinking that we know everything about staying safe yet in actual sense, we are ignorant…. For example, I was green about wearing a condom, I thought the moment I hold it and put it on him it is done, but the facilitators we had, and some other women showed us exactly how to safely wear it.

While Afiya, engaged in sex work two years, said that she might still “*accept to have unprotected sex because of the desire for much money”*, condoms became a non-negotiable for many participants. For instance, Gertrude, who had been engaged in sex work for five years, said she was more assertive since she attended the sessions and had been using condoms in all her sexual intercourses.

Gertrude: Previously, I would not be assertive and so I would say to myself that it was ok if I had missed using protection for some time, but when I received these sessions, I have never (Laughed) missed using a condom!

Women felt empowered to require their customers to wear a condom and refuse to have sex with them if the men did not want to, as exemplified in the response from Sanyu’s, who had been in sex work for over five years.

Sanyu: Even when the customer insists on having sex without a condom I cannot accept because they created awareness among us, even when they are offering me a lot of money you tell them no, life is more precious than money.

Agnes, engaged in sex work for two years, echoed similar thoughts. The message that being infected with the disease would have a higher financial cost than what a man would pay for unprotected sex convinced Agnes to refuse unprotected sex. Being given boxes of condoms made it easier for her to provide condoms to her customers when they did not have one.

Agnes: No condom, no deal! They told us to stop yearning for much money, because this will never pay for your hospital bills once infected. I would rather give in for someone with little money but with a condom.

Some women took the matter in their own hand to ensure that men wore condoms and that they did so properly as they were concerned that the clients might pierce the condom or not put it on if it was left up to them. Anitah, engaged in sex work for more than five years, discussed why she decided to use her own condoms and help her customers put on a condom. She added that she would not have sex with them if men did not want to wear it.

Anitah: I decided to help all my customers wear condoms, for instance, if a man puts on a condom by himself or decides not to wear it, I do not accept to have sex with him….I fear that he might pierce the condom if he wears it by himself….I am more confident about having protected sex when I help my customer put on a condom….I totally refuse condoms brought by customers because I do not trust their safety.

#### Female condom use

Female condoms were less frequently brought up and yielded mixed results in terms of their usefulness. For some, it was the first time they heard about female condoms and how to use them. Some women appreciated being introduced to female condoms and found them helpful, especially when customers refused to use male condoms. For instance, Flora, who had been engaged in sex work for six years, made sure she had female condoms readily available when men did not want to use condoms.

Flora: The majority of men come to us when they do not want to use condoms. So, what I do most times is to provide my own condoms. By the time they are undressing, I already have my condom for females with me.

Joyce, who recently started sex work, stated since she started using female condoms, she did not get “*itchy in her private parts*” anymore, potentially pointing to issues of yeast/infection she had experienced prior to female condom use. Others found female condoms impractical. They reported that putting it on *“consumed a lot of time”* and required one to *“keep holding the condom during sex so that it doesn’t slip off”.* Anitah went on to say that her colleagues also *“found it difficult to use due to the same problem [of having to hold it during sex]”* when she shared the information about female condoms.

Overall, WESW agreed that male condom was one of the important learning points for them and planned to continue using them with their customers. A theme that was dominant in their discussions was the concern around men not wearing the condoms properly, even when they agreed to do so. Notably, many women felt empowered to request men to wear condoms, and specifically to help them put on. On the other hand, female condoms were received with mixed reactions. While some women found them helpful and a safer option for men who prefer condomless sex, others thought they were impractical, and hence chose not to use them.

### Alcohol/drug consumption

One of the frequently mentioned benefits of the HIV risk reduction sessions was the focus on reduction of alcohol and drug consumption. Participants talked about the relationship between their alcohol/drug consumption and behaviors and situations that were putting them at risk. For instance, Esther, engaged in sex work for about 15 years, talked about how “getting drunk” might interfere with one’s ability to protect themselves from a customer infecting them with a disease.

Esther: You may get drunk, and someone picks you off the road…. There are instances when you get drunk and out of your mind, where a man could have sex with you and infects you with the disease. We were advised to minimize alcohol drinking or even doing away with it.

Another participant, Maria, working in sex work for five years, mentioned that customers might be more likely not to pay or “steal it” when she was drunk.

Maria: I was a drunkard and I used to take a lot of alcohol…. I used to go to a disco and first take waragi [local liquor], then take other kinds of alcohol so that by the time you go, you are already drunk. A man would at times reach the moment of giving you the money and he doesn’t give it to you, or even steal it after giving it to you together with your other money.

Kirabo, who had been engaged in sex work for about 10 years emphasized that drug and alcohol use might compromise one’s judgment and reducing drug use might allow someone to make informed decisions about their well-being.

Kirabo: I found out that if you always drink alcohol, marijuana, use “amayirungi” (khat), and shisha…You may end up doing inappropriate things because you are high on these …I learned that when one reduces drug use, they might end up making informed decisions.

Many participants talked about how they reduced their alcohol and/or drug consumption. Some reduced their alcohol intake while others quit completely. Barbara, who had been engaged in sex work for ten years, discussed how she reduced her alcohol consumption after attending the HIV risk reduction sessions.

Barbara: I used to drink alcohol without control before the sessions, however during sessions, I was advised to reduce the consumption…. I used to take about five or six bottles, but I now take one and then I sleep.

Viola, engaged in sex work for about five years, also talked about how she reduced her drug use and quit alcohol as a result of attending HIV risk reduction sessions.

Viola: I learned a lot because I used to take alcohol, drugs, and smoking pipes…. I would smoke pipe at least four times a day but at this time as we speak, ever since the Kyaterekera sessions.…I cannot say I quit the pipe completely since I now use it like once in a month. As for alcohol, I quit it completely.

Patience had been engaged in sex work for four years. She quit alcohol after attending HIV risk reduction sessions and reflected on the changes she experienced afterwards.

Patience: I was drinking a lot of alcohol! I could spend my nights in the bar but now I can spend the whole week without [drinking]…I can sleep and even if someone tells me about alcohol, I just tell them I am done with it. When I am quiet during my free time, I meditate upon how I used to drink a lot and the change I have encountered now….

For Anitah, who has been engaged in sex work for more than five years, the main motivation for quitting alcohol was to avoid contracting HIV.

Anitah: Currently I quit alcohol because I learned that one cannot easily stick to condom use when drunk hence a customer may take advantage of her. This increases the risk of contracting HIV/AIDS.

When asked about skills and knowledge they planned to use in the future, many of the women who identified reducing alcohol/drug consumption as a critical area of learning for themselves also mentioned that they would continue to stay away from using alcohol and other drugs. For instance, Mariam, engaged in sex work for over 10 years, mentioned that she foresaw herself continuing to reduce the consumption of *“intoxicating drugs”* as she observed *“a great improvement from the time I reduced their consumption”.*

In sum, many women appreciated receiving information about the potential risks of consuming alcohol and drugs, especially in the context of sex work. They discussed how they reduced or eliminated their alcohol or drug consumption and the benefits they experienced since then.

#### PrEP use

WESW also appreciated obtaining information about PrEP as a preventative method against acquiring HIV/AIDS. For instance, Joyce found it helpful to learn that *“in case you appropriately take PrEP tablets, you cannot easily acquire HIV*.” Afiya shared that she had been taking PrEP medication since she attended the sessions. This allowed her to feel safe when she found herself in situations where the customer wanted unprotected sex.

Afiya: Sometimes a man may bargain with a lot of money and lead you into unprotected sex. However, we were taught that in such a situation, we may take PrEP medication. From the time I attended the Kyaterekera sessions, I began taking my PrEP medication. I feel safe even when a customer insists on having unprotected sex.

Esther thought using PrEP was more preventative than using male condoms, especially as men may *“remove the condom in the middle of the act”.* While she struggled the first time she used PrEP, she did not feel any physical side effects once she followed the instructions given by the nurse.

Esther: During the sessions, we were encouraged to take these pills especially when we have customers, since it is hard to tell which customer who may infect you…. The first time I used it, I became dizzy because I had not eaten anything. Later alone I remembered that the nurses advised us to first have something to eat before we take the pill. Each time I use it when I have eaten something, I don’t face any challenge [side effects]. Using the pill is more preventative than a condom since a man might take off the condom and infect you with the disease.

Both Betty, engaged in sex work for a couple of years and Flora stated they would continue to take their PrEP “*medication”.*

Overall, information related to PrEP was appreciated by the participants, though identified by fewer participants when compared to condom use and alcohol consumption. WESW found it as a safe alternative to mitigate against the risk of clients refusing condom use or removing their condom during sexual intercourse.

#### "Handling” customers

One third of the participating women mentioned that learning *“how to handle customers”* was beneficial to them. Handling clients was described in terms of communicating in a friendly manner with the customers and was discussed mostly in the context of male condom use. For instance, both Sanyu and Betty talked about how they were able to *handle their customers well* and get them to eventually accept wearing a condom. Betty went on to add that if the customer resisted wearing a condom, she would *“find another way of satisfying him”*. Flora shared that she learned to calm her customer down when he was “excited or rough” during sexual intercourse to avoid the risk of condom bursting.

Other participants discussed other contexts in which this skill was beneficial to them. Viola described how learning to talk nicely to her customers allowed her to negotiate prices at times by providing the following example.

Viola: When I left the session, I decided to test out what I had learned about handling the customers well. When my first customer arrived, I called him very well and talked as if we had known each other before. He then asked me how much a “short” round cost and I told him that it costs seven thousand shillings. He replied that he had five thousand shillings and we negotiated until we agreed on seven thousand shillings. Since then, I have benefited a lot from using this skill!

Finally, Ruth mentioned that this skill allowed her to not have sex with her customers at times, but still get paid.

Ruth: During the sessions, we were trained that it is possible to meet with a customer, just have stories with him and then depart without even having sex with him. We have been able to make friends out of these customers, to the extent that a customer can send you money without having sex with him.

Overall, handling a client was discussed as a helpful strategy for safer sex, by some women in the sample, mainly by being able to convince customers to use condoms or in some cases avoiding sex altogether.

#### “Massaging” customers

Massaging was one of the alternatives provided to WESW as part of an activity called “Café Kyaterekera Menu” that listed a wide range of safer/lower risk sexual activities. Massaging, described by Gloria who has been engaged in sex work for about 15 years, as *“making men ejaculate without penetrating me”* was a strategy that was discussed during the HIV risk reduction as a safe alternative to unprotected sex that about one third of the women found beneficial. This included prolonged foreplay, kissing, touching and oral sex (using condoms). However, similar to female condom, other women mentioned massaging as a strategy that did not work for them. Kirabo was one of the participating women who found the strategy useful. She shared that in some cases, men may like it more and pay more when massaging was used.

Kirabo: We were given skills such as massage, for instance, you can agree with a customer to have sex with him but instead massage him without having penetrative sex. He could end up liking it and paying you more.

Ruth and Joyce said that before the sessions, they thought they would have to have sex with every customer. However, they learned different ways of massaging men and found that these worked when they tried with their customers. For instance, Joyce shared:

Joyce: I used the skill they taught us about massaging the man’s penis…. I went on well while conversing because he was my customer. He accepted and told me we would have sex the next time he was coming.

Flora and Anitah shared that massaging was a skill they would continue using. Specifically, Anitah shared that she would continue using massaging, but also *“have my condoms with me and help him wear it”* if a customer insisted on having penetrative sex.

Yet not all WESW found this strategy helpful. Barbara said she had not used that skill. Viola, Alice, and Afiya did not think massaging would work for them. Alice, who started sex work in her late teens, found it difficult to use and Afiya was skeptical that massaging would work with her customers because they paid to have sexual intercourse.

Afiya: What they taught us was about how we could handle customers in a way that doesn’t allow them to have sex with us. I thought it could be difficult because I have never experienced it; whoever buys you wants to have sex.

Viola was concerned that massaging was similar to “*live*” sex and that one would run the same risks of contracting diseases. She thought that she could get “*cancer of the mouth*” if engaged in “massag*ing men’s private parts*”, referring to oral sex.

Viola: I also failed to use that skill because it poses a threat of cancer. You can contract diseases since it is similar to having live sex…. For instance, if you start massaging a man including his private parts, you may end up getting cancer of the mouth…. So, if you wanted to get five thousand shillings you may end up incurring one million shillings or more in medical expenses!

Massaging was identified as a beneficial strategy to avoid penetrative sex by about one third of the sample. Similar to female condom, massaging also yielded mixed perceptions as some women thought that not having penetrative sex was an unrealistic expectation in their work.

## Discussion

In this article, we explored the perceived benefits of an evidence-based HIV risk reduction intervention informed by social cognitive theory and harm reduction approach among WESW. WESW identified five main areas that they benefited from, specifically condom use, alcohol consumption, PrEP use, “handling” customers, and “massaging” customers. While female condom use, PrEP use, and alcohol/drug consumption entailed individual choices; male condom use, handling customers, and massaging customers also involved navigating client expectations/requests in addition to individual choices for WESW to be able to change their behavior.

Information and skill-building around male condom use were the most frequently cited benefit of the intervention. The existing evidence shows that male condom use with paid clients is inconsistent among WESW [42, 43]. A study with WESW in Kampala, Uganda showed that 40% of WESW reported using condoms inconsistently with paying clients in the past month [42]. The most cited reason for inconsistent condom use was client’s preference, which entailed higher pay for WESW if they agreed to unprotected sex [42, 43]. Similar to these findings, WESW in our sample stated that prior to attending HIV risk reduction sessions, they did not consistently use male condoms, primarily because their clients did not want to. Insisting on using condoms meant that they would either accept a lower payment or risk losing the client altogether. However, women’s perspectives and practices around male condom use shifted after the intervention, mirroring the findings from a systematic review of behavioral interventions targeting condom use among WESW in SSA [13].

In our study, WESW appreciated learning about not only the consequences of not using male condoms, but also how to put one on their client. This is especially worth noting as it shows that many women had been engaged in sex work for many years without having access to this knowledge and skill together. Importantly, most WESW shared that HIV risk reduction sessions empowered them to negotiate condom use with their clients at the expense of losing their income because of the health consequences associated with unprotected sex. This feeling of “being empowered” aligns with the self-efficacy emphasis of the social cognitive theory that informs the intervention. As discussed in the literature, increase in perceived self-efficacy in negotiating and using condoms and decision-making in sexual relationships is a strong predictor of condom use [31, 32]. While whether WESW will be able to sustain this behavior change needs to be further examined, many voiced their intention to continue enforcing condom use. This is a promising finding as condom use has been identified as the most critical modifiable proximal determinant of both HIV infection and transmission among WESW [44].

In contrast to male condom use, WESW had mixed feelings about female condom use. Only a few women found female condoms helpful while others pointed out that they were not practical to use. This is in line with the mixed findings on female condom use among WESW. While some studies in SSA reported high levels of acceptability and utilization of female condom among sex workers [45], other studies documented low acceptability and utilization [46, 47]. For instance, a study conducted in four cities in India, Kenya, Mozambique and South Africa found that the percentage of WESW who reported ever using a female condom were 15.4% in Durban, 16.6% in Mombasa, Kenya, and 37.8% in Tete, Mozambique [46]. Similarly, a study conducted among WESW in Kenya reported high degree of substitution of the female condom for male condom [47]. Similar to our findings, facilitators of female condom use among WESW from across different settings include a perceived sense of negotiating power and control over safe sex, and the belief that female condoms are safer because they will not tear or be removed by a sex partner midway through sex [48, 49]. Barriers to female condom use include the risk of violent reactions from male sex partners, clients’ refusal or distrust of unfamiliar methods, difficulties during application and use, and high cost [50, 51]. Difficulties during application and use were also cited as main barriers to female condom use by WESW in our study.

Reduction in alcohol consumption was another benefit that many women mentioned. They made clear links about how their alcohol consumption contributed to their unsafe sex practices and undermined their ability to make healthy choices. These mirror existing evidence that show associations between alcohol consumption among WESW and high sexual risk behavior like unsafe sex, acts of violence and poor decision making, mental health problems, and the effect on treatment adherence for women living with HIV [52–54]. Many of the women who found information about alcohol and drug consumption helpful said that they significantly reduced or stopped their alcohol consumption as a result of the HIV risk reduction intervention and reported associated benefits. Adopting a harm reduction rather than abstinence approach in the curriculum may have been received more positively by WESW, further encouraging reduction in consumption. These findings are consistent with a South Africa study [16] and support the importance of incorporating a drug and alcohol consumption component in interventions targeting this population.

Similar to the findings in other qualitative studies conducted in SSA among WESW [51, 55, 56], WESW in our study believed that PrEP would give them the added protection, especially in situations in which they would not be able to use condoms with their clients or when condoms would burst or be removed. Among WESW who found PrEP-related content beneficial, PrEP was seen as a safe backup in case men would remove their condom during the sexual intercourse. Given that half of our sample lives with HIV and session 3 (which also focused on PrEP and PrEP enrolment) was one of the sessions with lowest attendance among this sample, with only 12 WESW attending this particular session (see Table 1), it is promising that about one third of our sample discussed PrEP content as useful. It is also important to note that PrEP was not brought up when women were asked about what content was not helpful to them. Qualitative studies conducted with WESW in SSA point to high acceptability of PrEP use [56–58]. In two randomized controlled trials in Zambia and Uganda with WESW, almost all participants in Zambia (91%) and 66% of participants in Uganda reported being “very interested” in daily oral PrEP [59]. A global systematic review of the PrEP care continuum among women who sell sex and/or use drugs also found high PrEP acceptability among WESW [60]. However, the existing literature identifies lower PrEP uptake and adherence among this population [60], documenting multi-level barriers have been documented by other studies conducted with WESW [60, 61]. While our qualitative findings suggest high acceptability, we did not specifically explore PrEP uptake and adherence in our qualitative study. Hence, even though WESW identified information about PrEP as a benefit from the study and expressed intention to use PrEP, whether their intentions materialized in actual behavior warrants further inquiry. However, our quantitative results from the clinical trial show that among the women who tested negative for HIV (n = 322), 91% (n = 317) expressed willingness to use PrEP and slightly over half of WESW not already on PrEP agreed to initiate PrEP (*n* = 158; 55%) [62]. The willingness to use PrEP was significantly associated with fewer years engaged in sex work and greater perceived social support from family. In addition, PrEP initiation was negatively associated with greater perceived social support from friends and positively associated with higher perceived stigma due to sex work among family members.

“Handling” a client was discussed in the context of negotiating safer sex during the HIV risk reduction sessions. WESW who thought that learning strategies, including communication skills, around how to handle a client was beneficial acknowledged that treating their “clients” nicely improved their ability to negotiate safer sex. Learning and practicing how to communicate with their clients, a key component of the social cognitive theory in HIV prevention, also led to other benefits, including negotiating better prices as well as “avoiding sex altogether, but still get paid”. This shows that WESW were able to internalize and apply this particular strategy to the different aspects associated with their work.

Finally, “massaging”—a local term to describe alternatives to penetration such as oral sex or masturbation- was identified as a beneficial strategy to avoid penetrative sex by about one third of WESW in our sample. Aligned with a harm reduction approach, massaging the client was one option among many introduced to WESW as a safe alternative to unprotected sex (e.g., shower with client, perform a strip tease). Women learned this skill from the intervention and their capacity to enact it speaks to their improved self-efficacy to identify and enact alternatives to risky sexual intercourse. Taken together, this ability demonstrates the skill of problem solving taught in the HIVRR intervention. However, while some women found it beneficial, others perceived this “massaging” to be unhelpful, primarily because they did not think it was realistic to expect their clients to be satisfied with it when men were there to have penetrative sex. In addition, one of the WESW shared her concern that certain types of massaging “may lead to cancer”. While this was only brought up by one participant, it may be hinting to existing misconceptions and/or myths that may require more deliberate efforts to dismantle.

Overall, WESW’s narratives reflect a boost in their confidence and self-efficacy as a result of the HIV risk reduction intervention across a range of risk behaviors and protective strategies. WESW pointed to the benefits of the information they received in the sessions as well as how they were able to more effectively communicate with their clients, two important aspects of increasing self-efficacy in HIV interventions informed by the social cognitive theory. In addition, goal setting, another critical component of the social cognitive theory, came across in WESW’s narratives when they discussed how they decided to reduce their alcohol use, negotiate condom use with their clients or refuse to engage in sex if the client did not want to wear a condom. On the other hand, the emphasis of the intervention on building support as a means to improve self-efficacy was not brought up by WESW as a perceived benefit. It is possible that WESW felt they were already embedded within a supportive network comprised of other WESW and hence did not see any added value. However, it is also possible that while building social support was a beneficial component of the intervention, other aspects of the program spoke to more pressing needs of this population.

In summary, our findings suggest that behavioral interventions targeting HIV prevention among WESW should adopt a holistic approach that not only cover traditional HIV prevention strategies, but also emphasize harm reduction techniques, including in the context of drug and alcohol use that is predominant in the lives of WESW. WESW also reported benefiting from both knowledge and skill building, pointing to the need for interventions to include not only information but also targeted skill building through role plays and visual aids that include practice opportunities. Finally, our sample included WESW across the HIV care continuum and WESW with a wide range of years spent in sex work. Our findings suggest that both groups could benefit from HIV risk reduction behavioral interventions.

### Limitations and future directions

Study findings need to be interpreted in light of study limitations. In this study, we used cross-sectional qualitative data collected upon completion of the intervention sessions, limiting our understanding of how participants’ perceptions about how they benefited from the HIV risk reduction intervention might have changed over time. In addition, we could not examine whether and how behavior changes reported by WESW were maintained over time. However, data analysis for 12-months follow-up data is ongoing. Findings may provide insights on the medium-term perceptions of the session benefits, as we as maintenance of these benefits over time. Finally, due to restrictions related to COVID-19 at the time of qualitative data collection, WESW in the treatment group were underrepresented.

## Conclusion

Despite these limitations, our study provides important insights into how WESW in hot spots in Uganda—a region yet underserved by best evidence interventions- may benefit from theory-informed evidence-based behavioral interventions targeting HIV risk reduction. Overall, our findings suggest that WESW found many components of our evidence-based intervention beneficial and described them as useful strategies that improved their ability to engage in safer sex and stay healthy. The fact that WESW identified other strategies beyond condom use as relevant and helpful in their work underlines the importance of adopting a comprehensive approach to behavioral interventions targeting HIV prevention even when combined with other interventions seeking to address the structural barriers, including financial, that undermine WESW’s ability to negotiate safer sex or transition out of sex work. In addition, behavioral interventions targeting HIV prevention should be informed by theory. WESW’s narratives suggest that incorporating the tenets of social cognitive theory and harm reduction approaches in HIV prevention among this population can result in risk behavior change.

## Supplementary Information

**Additional file 1.**Time 1 Qualitative Interview Guide.

## Data Availability

Data used in this analysis is available upon reasonable request, data access requests can be sent to any of the following Associate Deans—at Washington University’s Brown School. Provided the conditions outlined below are met, there should not be concern about data sharing. The team is open to data sharing provided the points outlined below, which were part of the study protocol, data sharing plan, and consenting process, are met. Siomari Collazo-Colón, JD, Associate Dean for Administration, Hillman Hall, Room 254, [o] 314.935.8675 [f] 314.935.8511, Brown School | Washington University in St. Louis, [e] scollazo@wustl.edu OR Fred M. Ssewamala, PhD [Study Co-PI], William E. Gordon Distinguished Professor, Associate Dean for Transdisciplinary Faculty Research, Professor of Medicine, Washington University School of Medicine, Goldfarb, Room 343, Brown School Washington University in St. Louis, [o] 314.935.8521 [e] fms1@wustl.edu. A formal research question is specified a priori. Names, affiliations, and roles of any other individuals who will access the shared data; The deliverable(s)—e.g., manuscript, conference presentation—are specified a priori; Proper credit and attribution—e.g., authorship, co-authorship, and order—for each deliverable are specified a priori. A statement indicating an understanding that the data cannot be further shared with any additional individual(s) or parties without the PI’s permission; IRB approval for use of the data (or documentation that IRB has determined the research is exempt). The requestors are expected to handle converting electronic formats (though the research team will consider converting to tab-delimited text format if possible). These conditions were prespecified in our study proposal, study protocol data sharing plan, and consenting and assenting process. Participants enrolled in the study are vulnerable women engaged in sex work, and over 40% of them living with HIV –both highly stigmatized. Thus, to protect this very vulnerable group, we stated in the consent form that only de-identified individual-level data may be shared outside of the research team and only upon completion of the conditions described above.

## Abbreviations

PrEP: Pre-exposure prophylaxis
SSA: Sub-Saharan Africa
STI: Sexually transmitted infection
WESW: Women engaged in sex work
