# Supplementary material for: "I expected little, although I learned a lot": perceived benefits of participating in HIV risk reduction sessions among women engaged in sex work in Uganda
Source: BMC Womens Health. 2022 May 13;22:162. doi: 10.1186/s12905-022-01759-1 (PMC9100307; doi:10.1186/s12905-022-01759-1)
Supplement: Supplementary file 1 — Additional file 1.Time 1 Qualitative Interview Guide. [file 12905_2022_1759_MOESM1_ESM.pdf]

HIVRR GROUP_TIME 1 (6 months)

[Interviewer Note: Please note the following information on the recorder.
- Cohort participant is in
- Study condition

INTRODUCTION *[TO BE RECORDED ON THE RECORDER ONCE YOU MEET WITH THE PARTICIPANT]*

[Interviewer Note: At the beginning of the interview, start the recording; state interviewer name, participant's ID number, time, date, and location of the interview.]

We would like to thank you once again for agreeing to complete the Kyaterekera project in-depth interview. We appreciate your interest and your willingness to invest your time and energy in the program.

We expect that the interview will take about an hour to 90 minutes. We can take a break along the interview so that you can get a drink or go to the restroom.

During the interview, we will ask a lot of questions about your experience with the Kyaterekera project. Please answer the questions as honestly and accurately as you can. There are no right or wrong answers. We just would like to better understand your experience with the program to be able to improve on it. Let us remind you that all your answers will be kept in strictest confidence and you can ask to skip any questions you don't feel comfortable answering. Your name will not be on the interview. Instead, we will give you a code number.

We will be tape recording the interview so that we can ensure we represent your answers accurately, but your information will be protected and only study staff will have access to this information.

Do you have any questions? Great! Let's get started!

1. Thank you again very much for participating in our project. Could you tell us a little bit about why you decided to participate?

- How did you initially hear of our project?
- What did you hope to learn from our project?
- What parts of the project sounded most appealing or most interesting to you?
- What parts of the project, if any, made you hesitate to take part?
- Who else did you discuss the project with as you were making your decision?
  - Why specifically those people?
  - What did they think about the project?

- What other health-focused projects have you participated in before?
  - Can you tell me what they were about?
  - How did you expect Kyaterekera to be similar or different to these other projects? And why?

Now, we would like to ask you a little bit more about the HIV risk reduction sessions you attended.

ATTENDANCE

2. Can you tell us about your experience attending the sessions?

- What were the things/factors that made it easier for you to attend the sessions?
- What were the factors that made it difficult for you to attend the sessions? (Interviewer note: Give participants time to think and answer. If the participant is having trouble, you can share these as examples: barriers regarding time management, childcare, sickness, transportation)
  - How did you overcome those barriers?

CONTENT

3. Overall, what did you think of what was covered in the sessions?

- What information covered in the sessions has been most helpful to you?
  - How have you been using them in your everyday life? Can you give us an example?
- What information covered in the sessions has been least helpful to you? Why?
- What skills covered in the sessions have been most helpful to you?
  - How have you been applying them in your everyday life?
    - Can you tell us about a time when you used them and felt like they worked really well?
    - Can you tell us about a time when you used them and felt like it didn’t go as well as you hoped?
  - Which skills do you foresee yourself continuing to use in the future? How?
  - What skills covered in the sessions have been least helpful to you? Why?

- How about sharing what you learned with your colleagues, friends, or other members of your family? What skills or knowledge have you shared and with who?
    - What did they think about what you shared with them?
- What other information or skills would you have wanted to see covered? Why?

FORMAT

4. What did you think of the way the sessions were delivered?

- What did you think about the group format?
  - What was most helpful? Why?
  - What was not so helpful? Why?
- What did you think about day and time of the sessions?
  - If it were up to you, when would you have these sessions?
- How about where the sessions were delivered?
  - What was most helpful? Why?
  - What was not so helpful? Why?
  - If it were up to you, where would you hold these sessions? Why?
- Can you tell us a little bit more about what you thought about the facilitators?
  - How were they in the way they facilitated the group session?
  - What did you think about how they conveyed the information?
  - What did you think about how they interacted with the group?
  - What was most helpful about how they conducted the sessions?
  - What was not so helpful?
  - If you were the facilitator, what would you do differently?

GENERAL

5. In what ways do you think these sessions would be helpful for other women in the area?

6. What recommendations do you have for us as we think about how to bring this program to many more women?

- *What would you change? (about content, facilitators, frequency, location) Why?*
- *What would you keep the same? (about content, facilitators, frequency, location) Why?*
- *How can we make it more self-sustaining?*

7. What else would you like us to know?
